# Supplementary material for: Conserved and divergent features of human mRNA decapping revealed by biochemical reconstitution
Source: Nat Commun. 2026 Apr 21;17:3697. doi: 10.1038/s41467-026-72177-2 (PMC13103063; doi:10.1038/s41467-026-72177-2)
Supplement: Supplementary file 1 — Supplementary Information [file 41467_2026_72177_MOESM1_ESM.pdf]

SUPPLEMENTARY INFORMATION

Conserved and Divergent Features of Human mRNA Decapping Revealed by Biochemical Reconstitution

Eric A.J. Simko[1], Sowndarya Muthukumar[2], Tanner M. Myers[1], Anna L. Valkov[1], and Eugene Valkov[1]

[1] National Cancer Institute, National Institutes of Health, Frederick, MD 21702, USA.
[2] Division of Molecular Hematology, Department of Laboratory Medicine, Lund Stem Cell Center, Faculty of Medicine, Lund University, Lund, Sweden.

Supplementary Table 1 | RNA substrates.

Name	Sequence	Label
**Decapping substrate (Me31B 3' UTR)**	GGGAGAAACGGAUAUGCCC UGUGUAACGCUAGUAUCUG AAAUAGAGUCUAAAUCGUGG AAUCAAUAAAAACAGAUGAA CUAUUUCAGAAAUCAAAAUU GUAUUAAAUAAAUGGAAAGC AAUUCAAUUUUUAAAGCGGC CGCAUUCUUAU	5' m7G[**γ**-32P]
**EMSA substrate (Rrp41 2xDE)**	GGGACCUCCGGAAACCGUA GAUUCCGGGCGGUCGGAGC CGCCGGGAGCUGUAGUUCU CCC	3' 6-FAM

Supplementary Table 2 | Protein constructs.

Protein	Description	Plasmid	N-term tag	C-term tag	Codon optimization	Notes
Hs DCP2(1-420)	full length	pnEA-NpM-DCP2(1-420)	MBP	6xHis	none	RefSeq NM_152624
Hs DCP2(1-245)	C-term IDR truncation	pnYC-NpM-DCP2(1-245)	MBP	n/a	none	RefSeq NM_152624
Hs DCP2(1-420)	full length	pnEA-NpM-DCP2(1-420)-2xStrepII	MBP	2xStrepII	none	RefSeq NM_152624
Hs DCP2(1-245)	C-term IDR truncation	pnYC-NpM-DCP2(1-245)-2xStrepII	MBP	2xStrepII	none	RefSeq NM_152624
Hs DCP1(1-135)	C-term IDR truncation	pnYC-NpM-DCP1(1-135)	MBP	n/a	none	RefSeq NM_018403
Hs DCP1(1-582)	full length	pLIB-His-DCP1(1-582)	6xHis	n/a	none	RefSeq NM_018403
Hs DCP1(1-582) TDm	L551R/I555S/F561R/L565S	pLIB-His-DCP1(1-582) (L551R/I555S/F561R/L565S)	6xHis	n/a	none	RefSeq NM_018403
Hs EDC4(1-1401)	full length	pACE-His-EDC4-2xStrepII	10xHis	2xStrepII	none	RefSeq NM_014329
Hs PNRC2(1-139)	full length	pnYC-NpM-PNRC2(1-139)	MBP	n/a	none	RefSeq NM_017761
Hs PNRC2(1-139)	full length	pnYC-NpM-PNRC2(1-139)-2xStrepII	MBP	2xStrepII	none	RefSeq NM_017761
Ce EDC4(520-843)	N-term truncation	pnYC-NpM-CeEDC4(520-843)	MBP	6xHis	none	RefSeq NM_171835
Ce EDC4(520-843)	N-term truncation	pnYC-NpM-CeEDC4(520-843)-2xStrepII	MBP	2xStrepII	none	RefSeq NM_171835
Ce DCP2(745-786)	N-term truncation	pnYC-CeDCP2(745-786)-SUMO3-2xStrepII	n/a	SUMO3, 2xStrepII	*E. coli*	*UniProt DCP2_CAEEL*
SUMO	full length	pnYC-SUMO-2xStrepII	none	2xStrepII	none	RefSeq NP_010798.1, residues 1-98
Sp Dcp2(1-504)	partial C-term truncation	pLIB-MBP-SpDcp2(1-504)-2xStrepII	MBP	2xStrepII	baculovirus	UniProt DCP2_SCHPO
Sp Dcp2(1-504)	partial C-term truncation	pnYC-NpM-SpDcp2(1-504)-2xStrepII	MBP	2xStrepII	baculovirus	UniProt DCP2_SCHPO
Sp Dcp2(1-242)	C-term IDR truncation	pnYC-NpM-SpDcp2(1-242)-2xStrepII	MBP	2xStrepII	baculovirus	UniProt DCP2_SCHPO
Sp Dcp1(1-127)	full length	pnEA-CvH-SpDcp1(1-127)	n/a	6xHis	*E. coli*	GenBank CAB69661.1

Supplementary Table 3 | Crystallographic statistics.

Space group	$P4_132$
**Unit Cell**	
Dimensions (Å)	
a, b, c	76.6, 76.6, 76.6
Angles (°)	
α, β, γ	90, 90, 90
**Data collection**	
Wavelength (Å)	1.0332
Resolution (Å)	44.2-1.42
R_{pim}	0.019 (0.446)
Mean $I/\sigma I$	24.7 (1.7)
Completeness (%)	100 (99.6)
Multiplicity	38.8 (35.6)
**Refinement**	
Resolution (Å)	44.2-1.42
No. reflections	15,054
R_{work} / R_{free}	14.8/17.0
No. atoms	443
**B-factors** ($Å^2$)	26.5
Protein	25.1
Ligand/ion	39.3
Water	35.4
**Ramachandran Plot**	
Favored (%)	100
Disallowed (%)	0
All-atom clash score	2.59
MolProbity score	1.56
**Root-Mean-Square Deviation**	
Bond lengths (Å)	0.009
Bond angles (º)	1.03

Values in parentheses are for the highest-resolution shell.
Ligand: one SO_4^{2-} ion

Supplementary Figure 1.

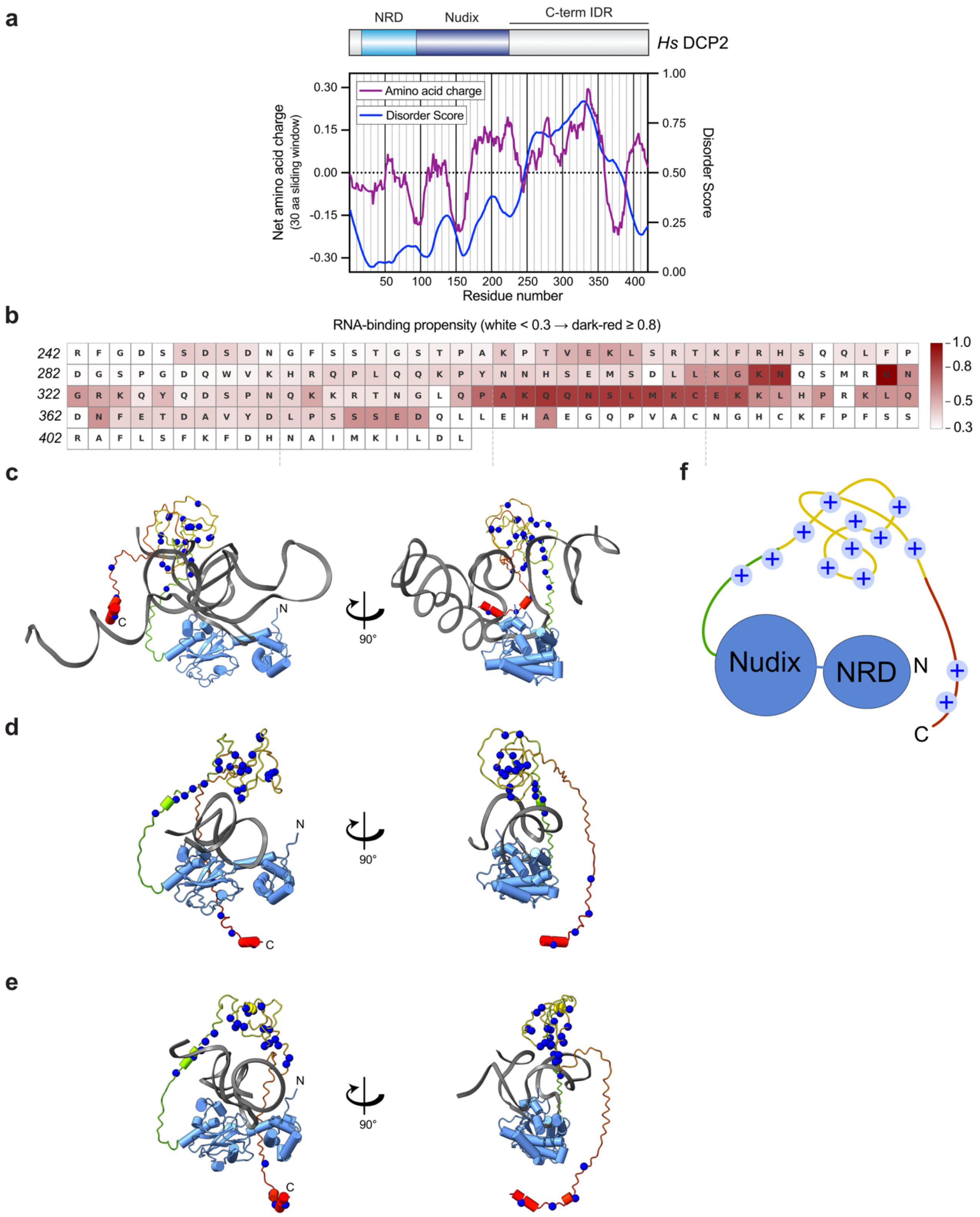

Supplementary Figure 1 | Charge and disorder distribution, per-residue RNA-binding propensity, and modeled RNA engagement of the *Hs* DCP2 C-terminal IDR.

(a) A linear schematic (top) shows the N-terminal regulatory domain (NRD), catalytic Nudix fold, and the C-terminal intrinsically disordered region (IDR) of *Hs* DCP2. Below, net amino-acid charge (magenta trace, left y-axis) and IUPred2A disorder score (blue trace, right y-axis) are plotted along the primary sequence using a 30-residue sliding window.

(b) Amino-acid positions are displayed in a Jalview-style grid that wraps every 40 residues (row labels indicate the first residue of each line). Each cell is color-ramped according to the flDPnn per-residue score, ranging from white for low propensity (<0.30) to dark red for high propensity (≥0.80); the continuous scale is shown on the right. Dashed vertical guides mark 40-residue intervals to aid orientation from the N-terminal residue (left) to the C-terminus (right). The representation highlights several clusters of elevated scores (e.g., 318-335 and 350-375), suggesting concentrated patches of RNA-contacting residues within this low-complexity region.

Panels **(c-e)** show two orthogonal views (0° and 90° rotation) of RoseTTAFold2NA predictions for full-length *Hs* DCP2 (N- and C-termini labeled) bound to three different RNAs; structured domains (NRD + Nudix) are colored light blue, the intrinsically disordered C-terminal region is rainbow-colored from green (proximal) to red (distal), and RNA is grey. All positively charged residues within the IDR are rendered as navy spheres.

(c) Me31B 3'-UTR RNA, the transcript used in the decapping assays.

(d) A 40-nt random RNA.

(e) The Rrp41 2×DE RNA used in EMSA experiments.

(f) Schematic summary of the recurring architecture: the folded Nudix and NRD domains form a rigid base (blue circles), whereas the IDR is conformationally unrestrained and presents a positively charged “noodle” (blue plus signs) that can contact a variety of RNA backbones. The model supports a role for the C-terminal IDR as an adaptable, conformationally flexible region that may guide substrates toward the catalytic core through charge-mediated recognition. It should be noted that the cartoon illustrates the spatial clustering of positively charged residues inferred from modeling; individual residue contacts are not defined.

Supplementary Figure 2.

a

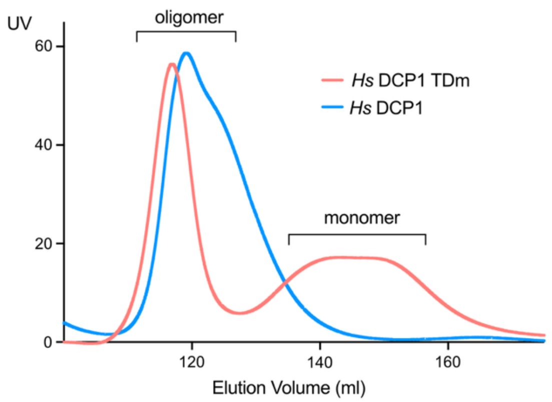

b

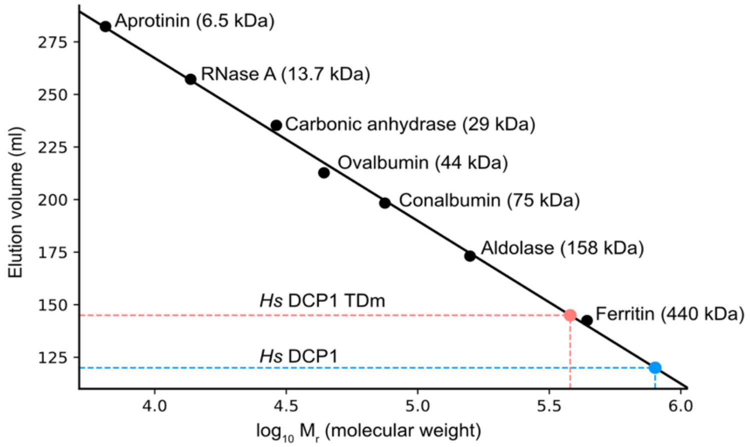

Supplementary Figure 2 | Size-exclusion chromatography (SEC) profiles of human DCP1 constructs reveal the oligomer-disrupting effect of the TDm mutations.

(a) UV absorbance (280 nm) is plotted against elution volume. Wild-type DCP1 (blue trace) elutes predominantly as an oligomeric species at 118-120 ml, consistent with its native trimeric assembly, with only a very minor tailing monomeric population. By contrast, the trimer-disrupting mutant (TDm; red trace) shows an additional broad monomer peak centered at ~145 ml in addition to the oligomer peak, indicating efficient disruption of higher-order assembly. Fraction identities (oligomer versus monomer) are annotated above the chromatogram; pooled fractions were verified by SDS-PAGE/Coomassie staining.

(b) Elution volumes of globular molecular-weight standards (aprotinin, RNase A, carbonic anhydrase, ovalbumin, conalbumin, aldolase, and ferritin; kDa indicated) were plotted against $\log_{10}(M_r)$ and fit by linear regression (solid line). The elution volumes of Hs DCP1 (blue) and the TDm mutant (red) are overlaid as dashed guides to indicate their apparent positions relative to the globular protein calibration. Because DCP1 contains extensive low-complexity/intrinsically disordered regions, it exhibits non-ideal (non-globular) hydrodynamic behavior and therefore does not map accurately onto the globular standard curve; accordingly, these apparent molecular-weight estimates should be interpreted qualitatively (assembly state/oligomerization) rather than as accurate molecular-weight estimates.

Supplementary Figure 3.

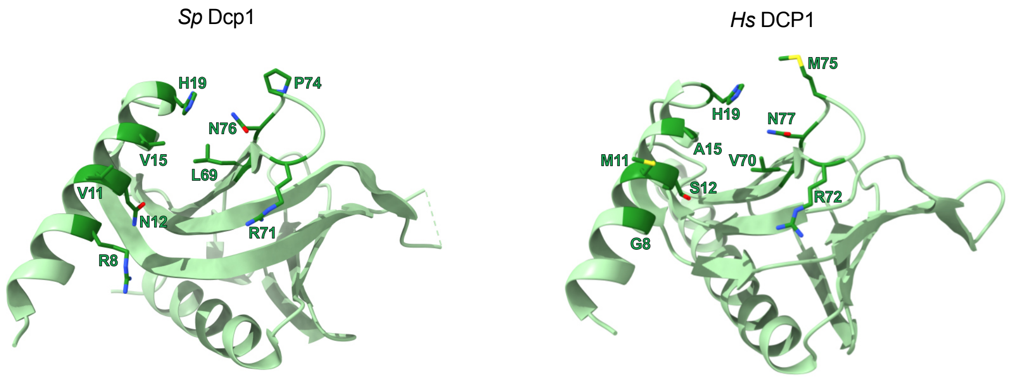

Supplementary Figure 3 | Comparison of DCP1 surface residues involved in the DCP2-DCP1 interface.

Interface residues are shown as dark green sticks on *Sp* Dcp1 as positioned in the Dcp2-Dcp1 interface (PDB: 2QKM) (left). Residues in positions corresponding to those involved in the yeast interface are shown as dark green sticks on *Hs* DCP1, as positioned in the AlphaFold Multimer prediction of the *Hs* DCP2-DCP1 complex (right).

Supplementary Figure 4.

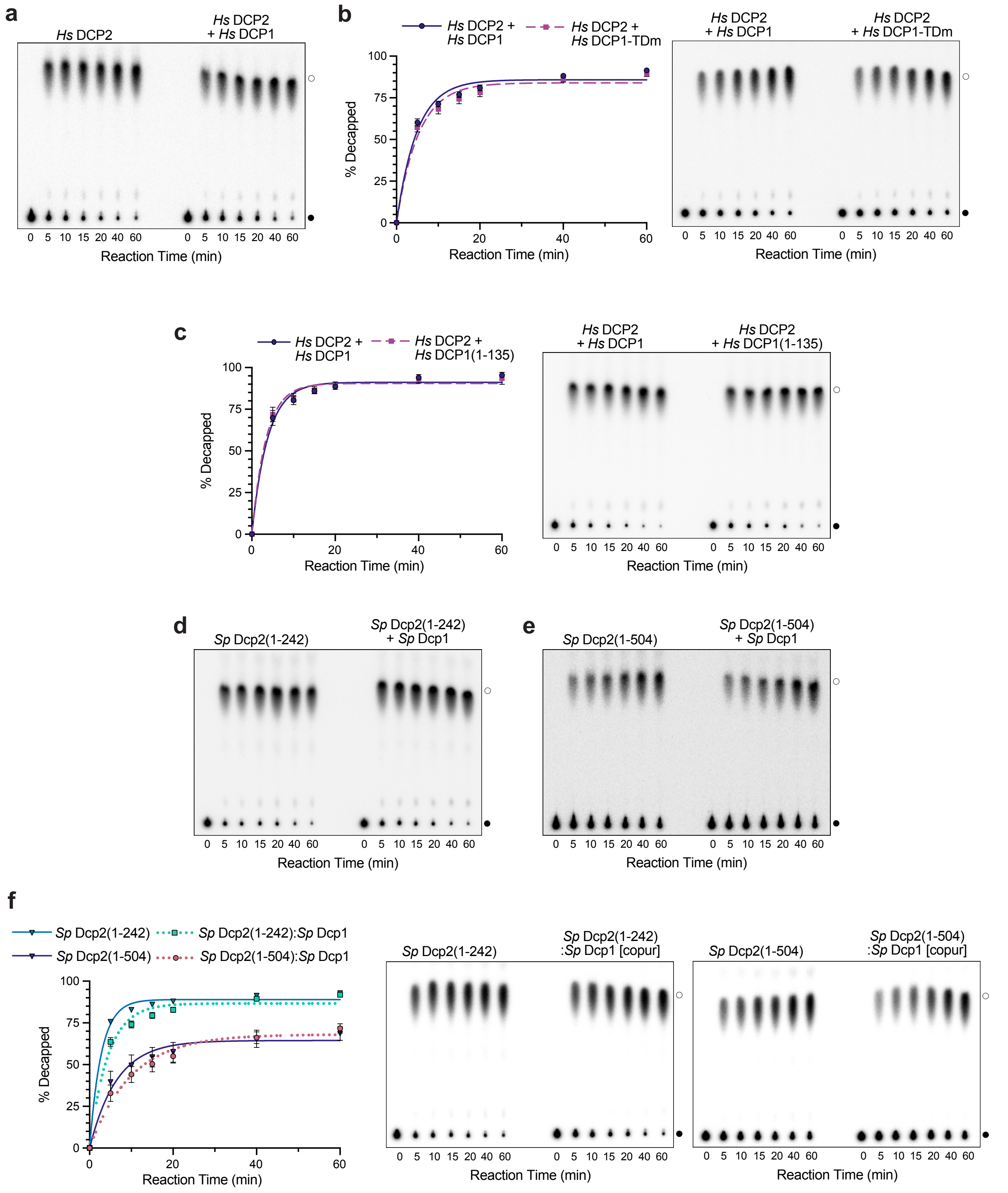

Supplementary Figure 4 | Influence of DCP1 variants on the decapping activity of DCP2 constructs with C-terminal truncations or oligomer-disrupting mutations.

(a) Full-length *Hs* DCP2 hydrolyses the mRNA cap with or without equimolar *Hs* DCP1. Reactions (0-60 min) were quenched, resolved by TLC; substrate (●) and m^7GDP product (○) positions are indicated.

(b) Comparison of wild-type *Hs* DCP1 and the trimer-disrupting mutant (*Hs* DCP1-TDm) on *Hs* DCP2 activity. Left, mean ± SEM (% decapped, n = 3) with single-exponential fits; right, representative TLCs.

(c) Effect of deleting the DCP1 trimerization/C-terminal domain. Activity of *Hs* DCP2 was monitored in the presence of full-length *Hs* DCP1 or the N-terminal EVH1-only construct *Hs* DCP1(1-135); data are plotted as in (b) (n = 3) with a representative TLC on the right.

(d) *Sp* Dcp2 catalytic core [*Sp* Dcp2(1-242); C-terminal IDR removed] decaps RNA in the absence or presence of equimolar *Sp* Dcp1. The representative TLC time course is shown.

(e) Same experiment as (d) using a longer *Sp* construct that retains part of the IDR [Sp Dcp2(1-504)].

(f) Direct comparison of the isolated *Sp* Dcp2 constructs (solid traces) with stoichiometric, co-expressed *Sp* Dcp1/2 complexes (dashed traces). Left, kinetic plots (mean ± SEM, n = 3) with single-exponential fits; right, representative TLCs for each condition.

Supplementary Figure 5.

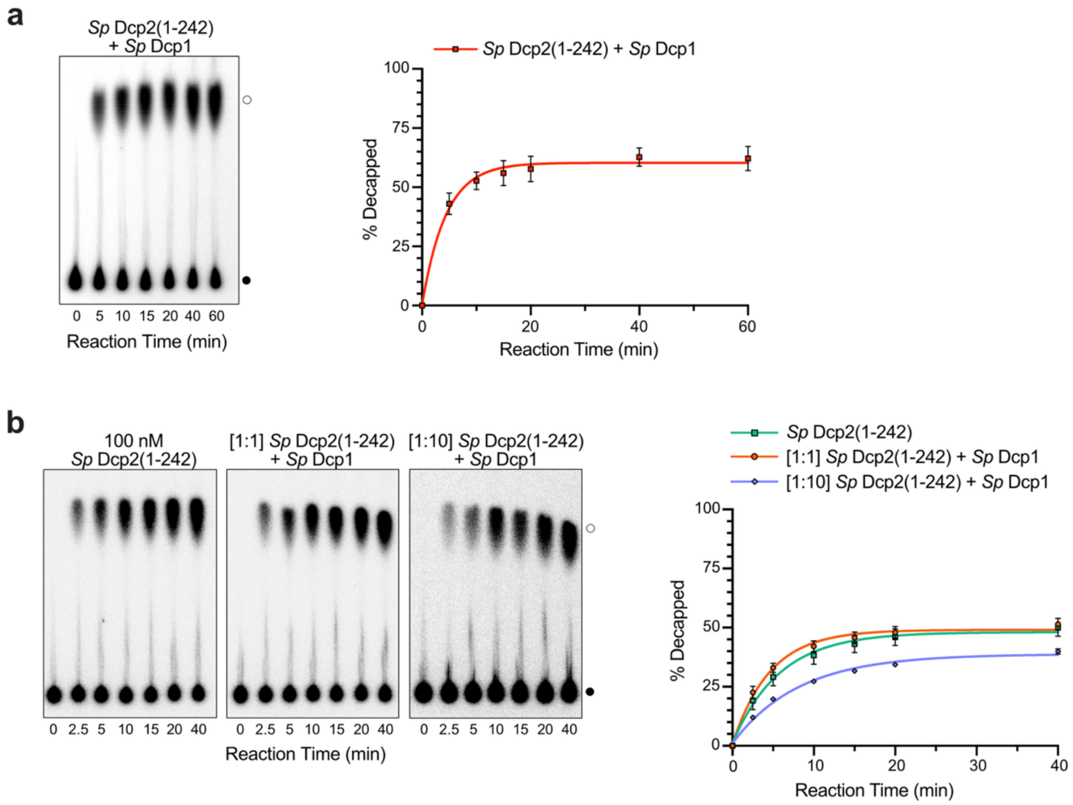

Supplementary Figure 5 | The effect of *Sp* Dcp1 on Dcp2 decapping in the presence of magnesium metal ion cofactor.

(a) Decapping by a pre-mixed *Sp* Dcp2(1-242)/Sp Dcp1 complex (200 nM each) was monitored for 0-60 min. Radiolabeled substrate (●) and m^7GDP product (○) positions were resolved by TLC and quantified. Product formation (right, red symbols) is plotted as % decapped RNA; points are mean ± SEM (n = 3) and the curve is a single-exponential fit.

(b) TLC (left) and corresponding kinetic analysis (right) showing mRNA cap hydrolysis by 100 nM *Sp* Dcp2(1-242) alone (green), in the presence of an equimolar amount of *Sp* Dcp1 (1:1, orange), or with a 10-fold molar excess of Dcp1 (1:10, blue). Reactions were quenched at the indicated times (0-40 min) and analyzed as in (a). Symbols represent mean ± SEM from three independent experiments; curves are single-exponential fits.

All reactions were carried out in buffer containing $MgCl_2$ as the sole source of divalent cation; no Mn^{2+} was added.

Supplementary Figure 6.

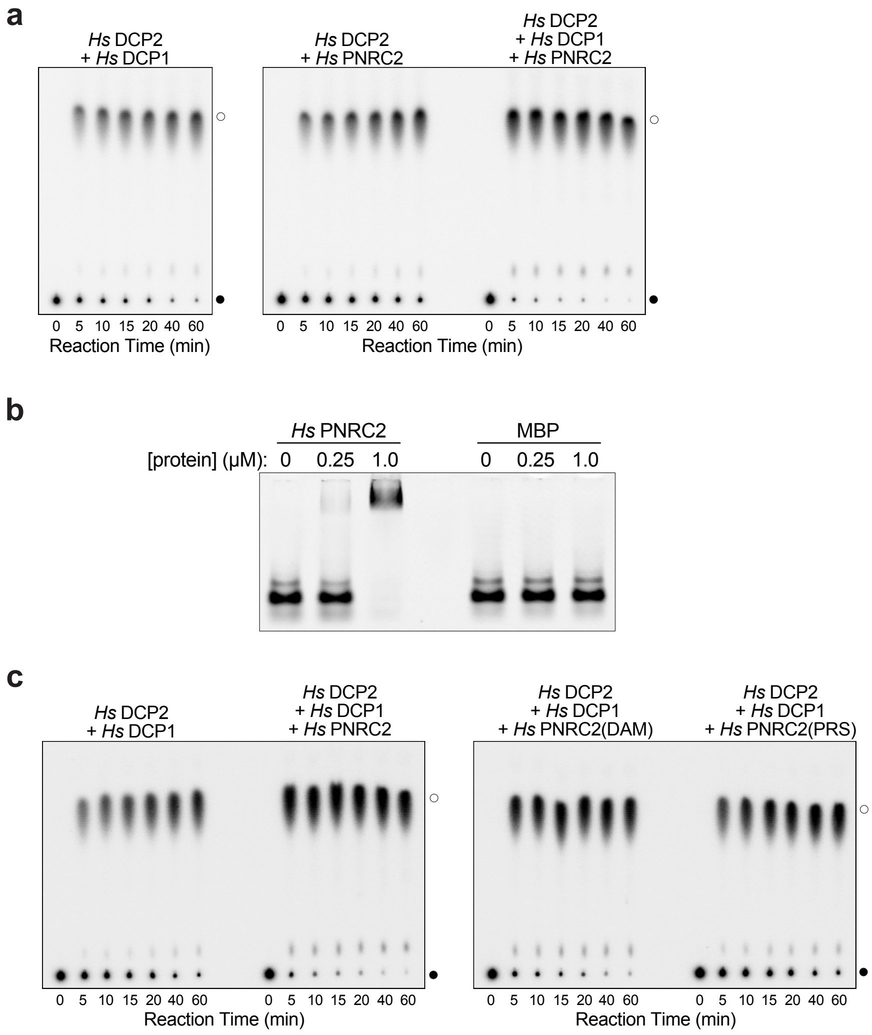

Supplementary Figure 6 | Influence of *Hs* PNRC2 on *Hs* DCP2 decapping and its intrinsic RNA-binding activity

(a) Decapping reactions showing the effect of *Hs* PNRC2 on the activity of *Hs* DCP2 in the presence and absence of *Hs* DCP1. Aliquots were quenched at the indicated times (0-60 min) and resolved by TLC. Filled (●) and open (○) circles mark substrate and m^7GDP product, respectively.

(b) Electrophoretic mobility-shift assays (EMSA) with a 5′-labeled Rrp41-2×DE RNA probe. Increasing concentrations of *Hs* PNRC2 (0-1.0 μM) produced a dose-dependent retardation of the probe, whereas maltose-binding protein (MBP) at identical concentrations had no effect. Representative gel from three independent experiments.

(c) Full-length *Hs* PNRC2 or the indicated synthetic peptides corresponding to the decapping-activator motif [PNRC2(DAM)] or the proline-rich segment [PNRC2(PRS)] were added (equimolar) to DCP1-stimulated decapping reactions. TLC time courses were performed and displayed as in (a).

Supplementary Figure 7.

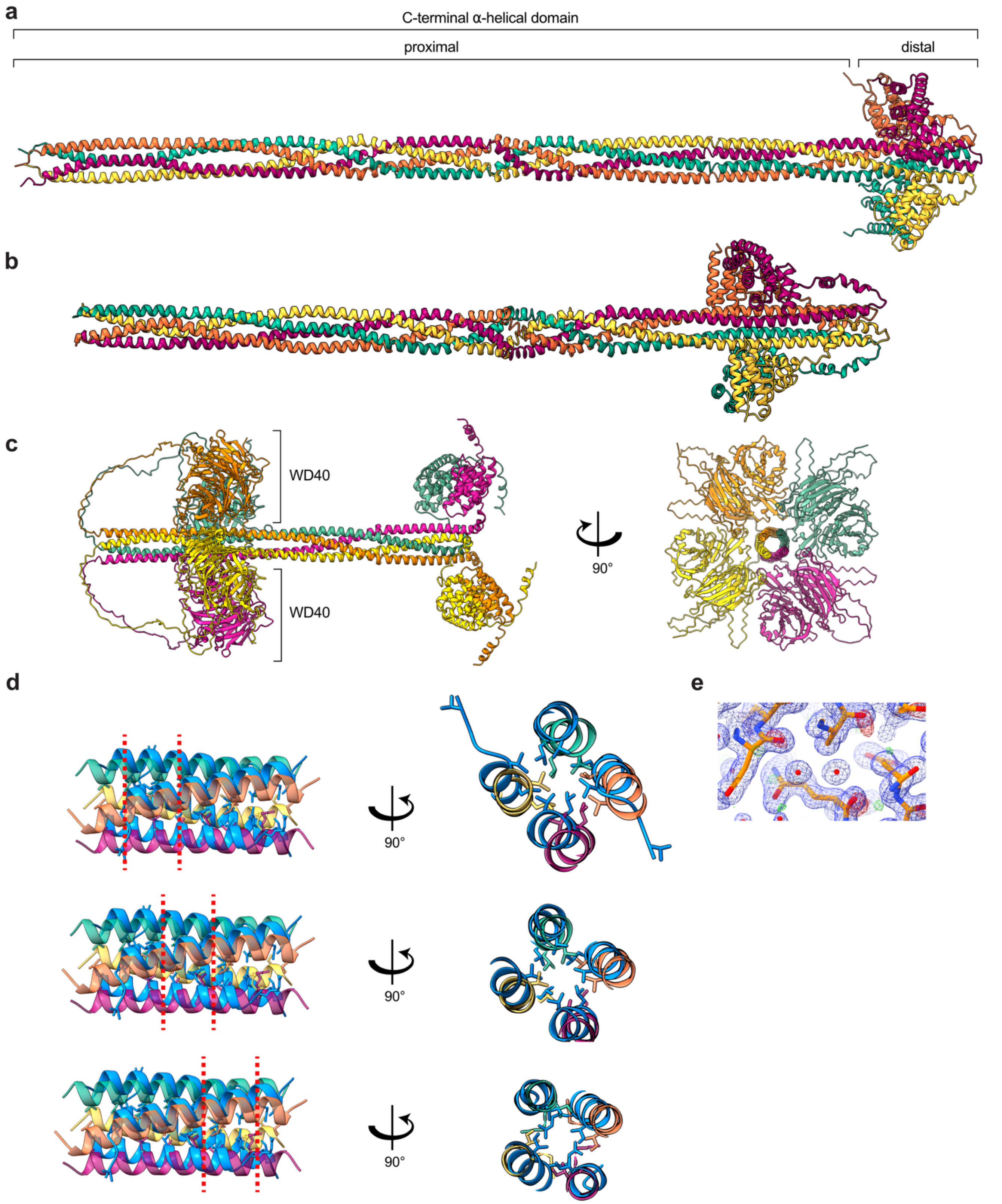

Supplementary Figure 7 | AlphaFold-Multimer models reveal a conserved tetrameric coiled-coil scaffold in EDC4 homologs.

(a) *Hs* EDC4 C-terminal α-helical region (residues 945-1401). Four protomers, each rendered in a different color, assemble into an extended parallel four-helix bundle. The segment is annotated as "proximal" (nearest the N-terminal WD40 domain, not modelled here) and "distal" (extreme C-terminus).

(b) *Arabidopsis thaliana* EDC4 C-terminal α-helical region (residues 915-1344) forms an analogous tetramer.

(c) Full-length *Ce* EDC4 adopts the same organization: the long coiled-coil core is preserved, while the N-terminal WD40 propellers (compact globular domains at the right) project from the proximal end of the stalk. The WD40 domains are flexibly linked to the coiled-coil stalk, and their orientation is stochastic. Left, side view highlighting two diametrically opposed WD40 domains (brackets); right, 90° rotation showing the four-fold symmetry of the propeller ring encircling the central rod.

(d) Validation of the predicted interface. Longitudinal cut-away views (left) and corresponding end-on cross-sections (right) compare the crystal structure of the isolated *Ce* EDC4(520-552) peptide (blue) with the equivalent region from each protomer in the AlphaFold-Multimer model (green, magenta, yellow, orange). Red dashed lines indicate the positions of the sections. The excellent superposition across three independent slices supports the register and packing geometry of the predicted tetrameric coiled-coil.

(e) A representative view of the electron density and the protein model. 2Fo–Fc map is shown as a blue mesh, at a contour level of 1.0 σ. Fo–Fc maps are shown in green (positive) and red (negative) mesh, at a contour level of 3.0 σ and −3.0 σ, respectively. The protein model is shown in stick representation and the ordered water molecules are shown as red spheres.

Supplementary Figure 8.

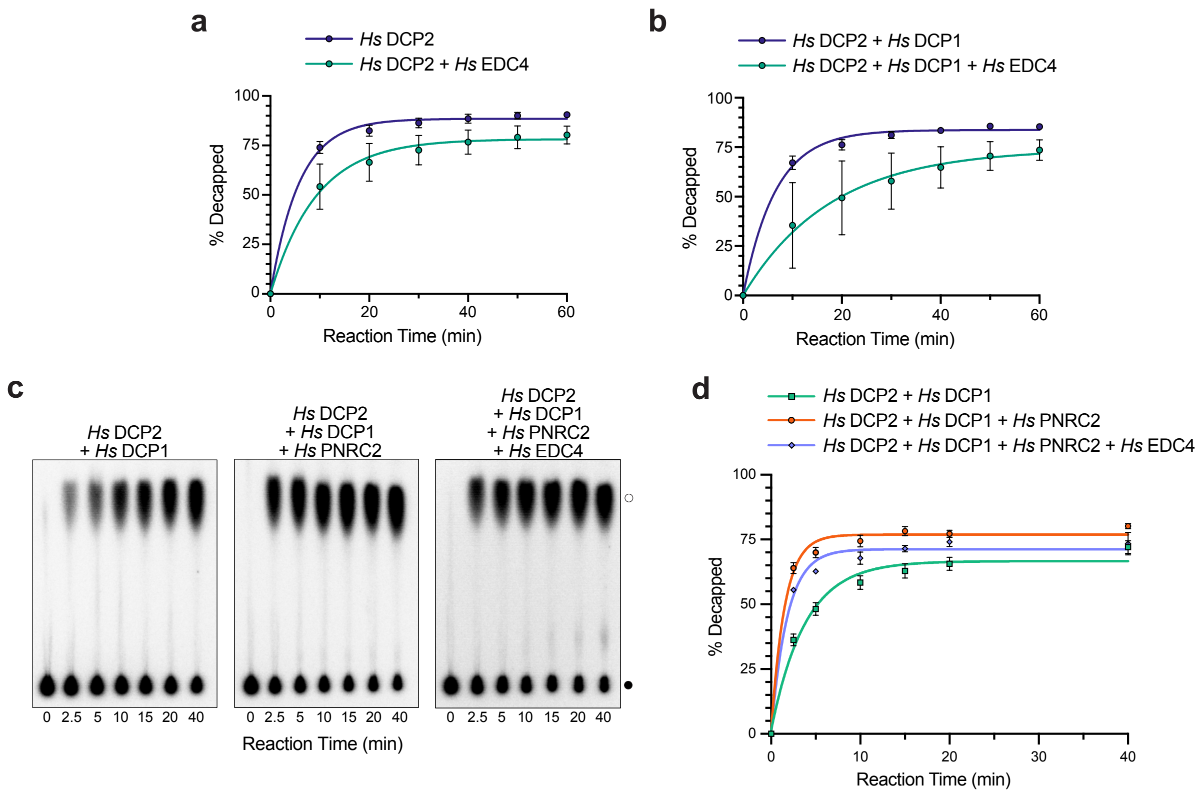

Supplementary Figure 8 | *Hs* EDC4 dampens *Hs* DCP2-mediated decapping in vitro and counteracts *Hs* PNRC2-driven stimulation.

(a) Cap-hydrolysis by full-length *Hs* DCP2 was monitored in the absence (purple) or presence of equimolar recombinant *Hs* EDC4 (teal). Reactions were quenched at the indicated times (0-60 min), resolved by PEI, and quantified. Symbols show mean ± SEM (n = 2); curves are single-exponential fits.

(b) Kinetic analysis of *Hs* DCP2 with equimolar *Hs* DCP1 (purple) versus the same mixture supplemented with equimolar *Hs* EDC4 (teal), plotted and analyzed as in (a) (n = 2).

(c) Left, DCP2 + DCP1; center, DCP2 + DCP1 + PNRC2; right, DCP2 + DCP1 + PNRC2 + EDC4. Aliquots (0-40 min) were processed as above. Closed (●) and open (○) circles denote substrate and m^7GDP product, respectively.

(d) Percent decapped RNA is plotted for DCP2/DCP1 (green), DCP2/DCP1 + PNRC2 (orange), and DCP2/DCP1 + PNRC2 + EDC4 (blue). Data are mean ± SEM (n = 3) with single-exponential fits.
